# Identification of gross deletions in *FBN1* gene by MLPA

**DOI:** 10.1186/s40246-018-0178-y

**Published:** 2018-10-04

**Authors:** Hang Yang, Yanyun Ma, Mingyao Luo, Kun Zhao, Yinhui Zhang, Guoyan Zhu, Xiaogang Sun, Fanyan Luo, Lin Wang, Chang Shu, Zhou Zhou

**Affiliations:** 1 State Key Laboratory of Cardiovascular Disease, Beijing Key Laboratory for Molecular Diagnostics of Cardiovascular Diseases, Diagnostic Laboratory Service, Fuwai Hospital, National Center for Cardiovascular Diseases, Chinese Academy of Medical Sciences and Peking Union Medical College, Beijing, 100037 China; 2 State Key Laboratory of Cardiovascular Disease, Center of Vascular Surgery, Fuwai Hospital, National Center for Cardiovascular Diseases, Chinese Academy of Medical Sciences and Peking Union Medical College, Beijing, 100037 China; 3 Department of Cardiovascular Surgery, Xiangya Hospital Central South University, Changsha, 410008 Hunan China

**Keywords:** Marfan syndrome, MLPA, *FBN1* gene, Deletion

## Abstract

**Background:**

Marfan syndrome (MFS) is an autosomal dominant connective tissue disorder caused by mutations in the *FBN1* gene. Approximately 90% of classic MFS patients have a *FBN1* mutation that can be identified by single-gene sequencing or gene-panel sequencing targeting *FBN1*. However, a small proportion of MFS patients carry a large genomic deletion in *FBN1*, which cannot be detected by routine sequencing. Here, we performed an MLPA (multiplex ligation-dependent probe amplification) test to detect large deletions and/or duplications in *FBN1* and *TGFBR2* in 115 unrelated Chinese patients with suspected MFS or early-onset aneurysm/dissection.

**Results:**

Five novel large deletions encompassing a single exon or multiple exons in the *FBN1* gene were characterized in five unrelated patients, of which four were proven by Sanger sequencing, and the breakpoints were identified. Three of them met the revised Ghent criteria when genetic results were not available, and the other two patients were highly suspected and diagnosed with MFS until the *FBN1* deletions were identified.

**Conclusions:**

Our finding expands the mutation spectrum of large *FBN1* deletions and emphasizes the importance of screening for large *FBN1* deletions in clinical genetic testing, especially for those with classic Marfan phenotype.

**Electronic supplementary material:**

The online version of this article (10.1186/s40246-018-0178-y) contains supplementary material, which is available to authorized users.

## Background

Marfan syndrome (MFS) is a connective tissue disorder with high clinical heterogeneity, mainly involving ocular, skeletal, and cardiovascular systems, with an estimated prevalence of 1:3000–1:5000 [1]. A large proportion of patients have visible signs, such as tall and slender stature, arachnodactyly, chest deformity, and scoliosis. Most patients have rapidly progressive myopia, and approximately 60% of affected individuals have ectopia lentis. However, cardiovascular abnormality might be the only defect in some MFS patients that is insidious and fatal.

MFS is caused by mutations in the *FBN1* gene, which is located on chromosome 15q21.1 and encodes a 320-kDa extracellular matrix glycoprotein fibrillin-1 [2, 3], a major component of microfibrils. So far, more than 2500 mutations (HGMD Professional 2018.1 total) have been identified throughout *FBN1*, while missense mutations are the most common type [4, 5]. Sanger sequencing of *FBN1* and panel sequencing including *FBN1* as well as a number of other genes associated with inherited aortopathies are commonly used to identify mutations [6]; however, both of these methods have a limitation for detecting *FBN1* large deletions (del) or duplications (dup), which have been reported in up to 7% of MFS patients [7].

Additionally, Loeys-Dietz syndrome (LDS), another inherited connective tissue disorder, which is caused mostly by *TGFBR1* and *TGFBR2* mutations, is often clinically indistinguishable from MFS [8]. However, up to now, no large genomic rearrangements in *TGFBR1* or *TGFBR2* have been reported in patients with aortic aneurysm/dissection and LDS features.

In this study, we performed a multiplex ligation-dependent probe amplification (MLPA) testing of *FBN1* and *TGFBR2* in 115 unrelated Marfan or early-onset aortopathy patients that were previously proven to be negative in a panel testing involving 15 genes associated with inherited aortopathy.

## Results

**Fig. 1:**
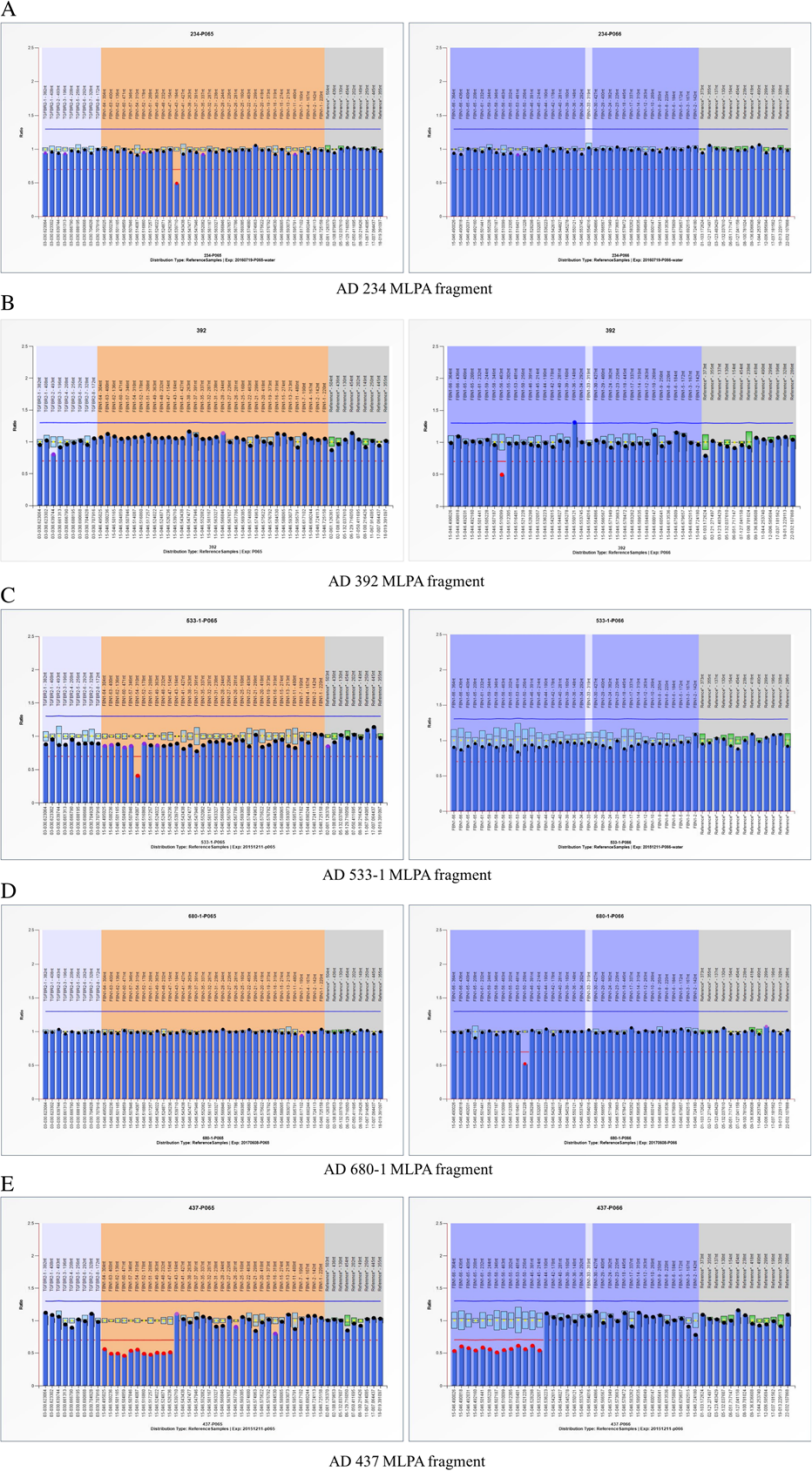
Results of semiquantitative MLPA. The results of MLPA for five patients. **a** Reduced relative peak areas of *FBN1* exon 43 for patient AD234. **b** Reduced relative peak areas of *FBN1* exon 56 for patient AD392. **c** Reduced relative peak areas of *FBN1* exon 54 for patient AD533-1. **d** Reduced relative peak areas of *FBN1* exon 50 for patient AD680-1. **e** Reduced relative peak areas of *FBN1* exon 44–66 for patient AD437

A total of 115 patients with suspected MFS or early-onset aortic aneurysm/dissection, who had a negative result in a 15-gene panel testing, were included in this study and evaluated for gross deletions and duplications in *FBN1* and *TGFBR2* gene by MLPA assay. The baseline clinical characteristics are summarized in Table 1. Of all patients, 19 were classic MFS, which referred to those who met the Ghent criteria independent of genetic results, and 43 were suspected MFS, which referred to those with some positive signs (either aortic dilation or positive family history AND systemic score ≥ 3) but not meeting the criteria yet. Almost half of the patients had no other systemic abnormality except for aortic events. Five novel large deletions encompassing a single exon or multiple exons in the *FBN1* gene were identified in five unrelated patients (Fig. 1, Table 2). Patients AD234, AD392, AD533-1, and AD680-1 harbored *FBN1* deletions of exon 43, exon 56, exon 54, and exon 50, respectively, while patient AD437 had a large deletion encompassing exons 44–66 in *FBN1*. These data had been submitted to ClinVar (ClinVar accessions SCV000804313-000804317).

**Table 1:** The baseline clinical characteristics of the 115 unrelated patients

Characteristics	Statistics (*n* = 115)
Age (years)	29.4 ± 14.9
Male gender	87 (75.7%)
Primary diagnosis	
Marfan syndrome	19 (16.5%)
Suspected Marfan syndrome	43 (37.4%)
Thoracic aortic aneurysm and dissection	53 (46.1%)

Values are presented as mean ± SD or *n* (%)

**Table 2:** Overview of cases with large deletions in *FBN1* gene

Patient No.	Age (y)	Deletion breakpoints	Deletion (*FBN1* exon affected)	Phenotype
AD234	24	g.48749026-48753819	*FBN1*: exon 43	Classic MFS
AD392	38	g.48724560-48722281	*FBN1*: exon 56	Classic MFS
AD533-1	5	g.48727672-48726338	*FBN1*: exon 54	Suspected MFS
AD680-1	14	g.48734801-48730690	*FBN1*: exon 50	Suspected MFS
AD437	37	NA	*FBN1*: exon 44–66	Classic MFS

All nucleotide positions are represented in relation to the human genome reference sequence (GRCh37/hg19), and position + 1 corresponds to the first nucleotide of the *FBN1* reference sequence (GenBank NC_000015.9) at the genomic DNA (g) level

*NA* not available

**Fig. 2:**
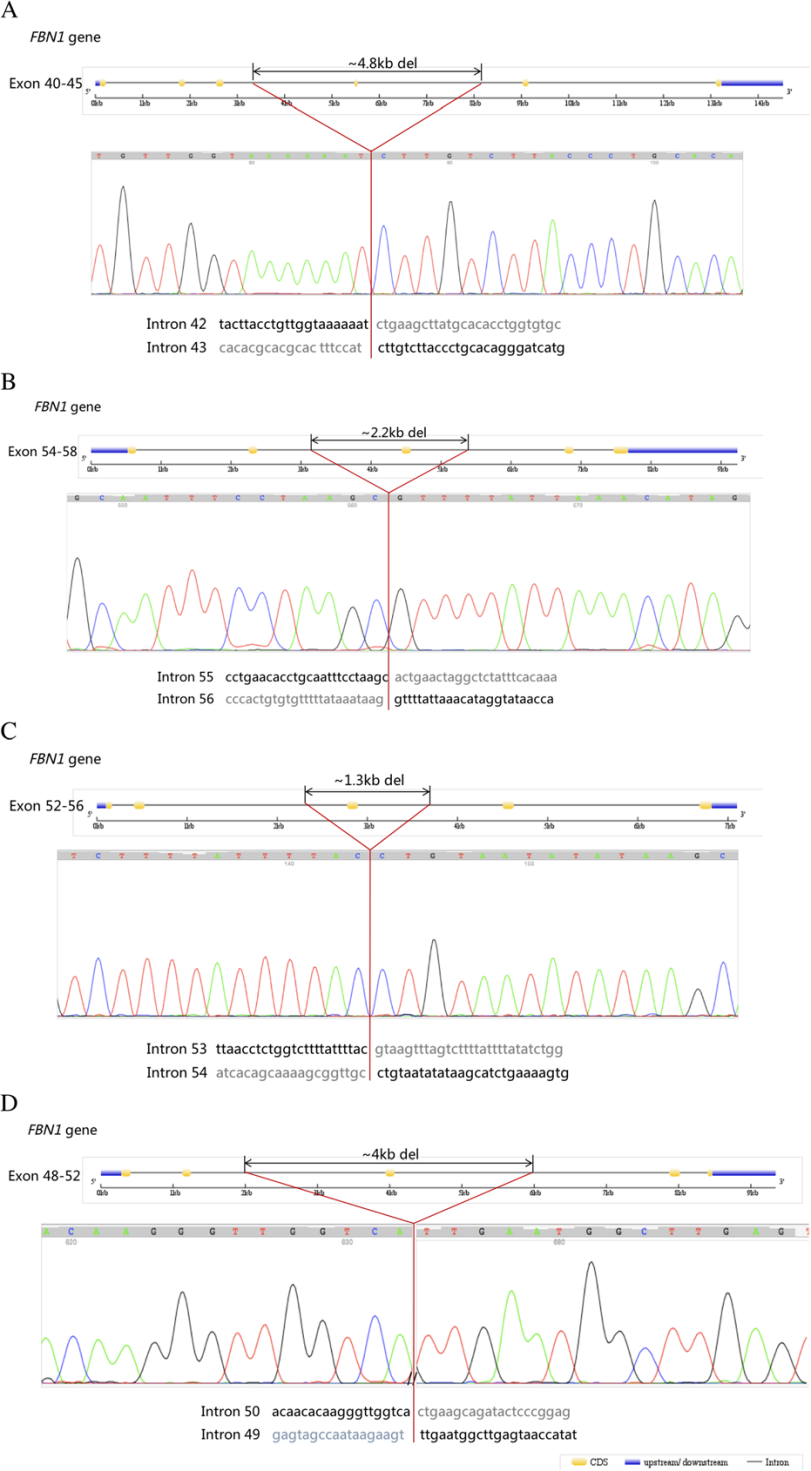
Sequences of PCR products spanning the breakpoint junctions of the four single exon deletions. **a** ~ 4.8 kb deletion encompassing exon 43 in patient AD234. **b** ~ 2.2 kb deletion encompassing exon 56 in patient AD392. **c** ~ 1.3 kb deletion encompassing exon 54 in patient AD533-1. **d** ~ 4.0 kb deletion encompassing exon 50 in patient AD680-1

**Fig. 3:**
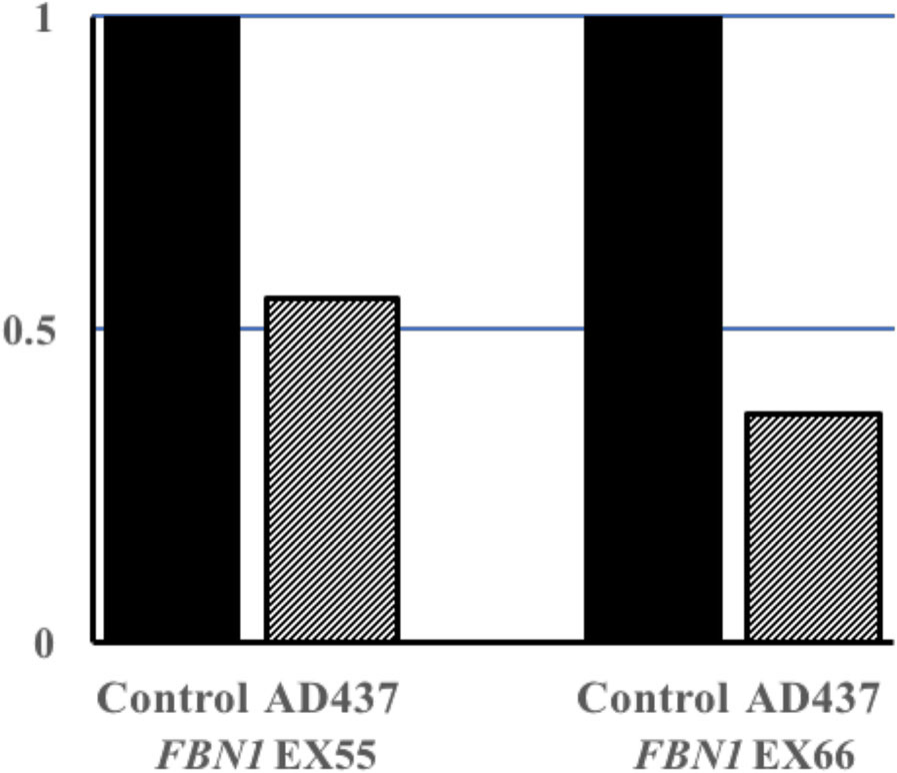
Verification of gross deletions in AD437 by quantitative PCR. The bar graph shows the relative ratio of DNA from AD437, indicating the presence of a heterozygous deletion in the region

To detect the breakpoints of deletions, we performed a long-range PCR followed by Sanger sequencing. Finally, the four single-exon deletions were all confirmed, and the breakpoints were found (Fig. 2). Regrettably, the deletion in AD437 could not be verified by the same method, since the mutated allele did not amplify well. Hence, we performed a quantitative PCR instead. Figure 3 shows that the quantity of genomic DNA from the proband amplified by primer pairs targeting exon 55 and exon 66 was half of that in the control samples, suggesting the true presence of a heterozygous deletion in this region.

All five *FBN1* large deletion carriers had multiple system deformities. Information on the clinical manifestation of the disease and family history is summarized in Table 3. Three patients (AD234, AD392, and AD437) were classic MFS, while the other two (AD533-1 and AD680-1) both had a systemic score of 6 but did not meet the criteria yet when genetic results were not available, probably due to their young ages. Combined with genetic results, these two patients were eventually diagnosed with MFS. Notably, patient AD437 had a gross deletion involving the last 23 exons, but there was no significant difference in the severity of clinical phenotypes when compared with the other four single-exon deletion carriers.

**Table 3:** The information of patients’ clinical manifestation and family history

	Patients				
	AD234	AD392	AD533-1	AD680-1	AD437
Age (y)	24	38	5	14	37
Gender	Male	Female	Male	Male	Female
Height (cm)	178	167	120	180	177
Weight (kg)	70	58	23	52	71
Cardiovascular system					
Aortic diameter (cm)	5.6	3.5	3.3	3.2	4.7
*Z*-score	8.8	2.0	6.8	2.1	6.2
Aortic dissection	Y	N	N	N	Y
Skeletal system					
Pectus carinatum deformity	Y	NA	Y	N	Y
Wrist and thumb signs	Y	Y	Y	Y	Y
Scoliosis or thoracolumbar kyphosis	N	N	N	Y	N
Joint hypermobility	NA	Y	N	N	N
Reduced upper segment/lower segment ratio AND increased arm/height	N	N	NA	Y	N
Hindfoot deformity	NA	Y	N	N	NA
Ocular					
Ectopia lentis	N	Y	N	N	NA
Myopia/strabismus	Y	Y	Y	Y	N
Other features					
Skin striae	N	Y	N	N	Y
Family history	Y	NA	N	N	Y

*Y* presence of criterion, *N* absence of criterion, *NA* not available

## Discussion

Marfan syndrome has a highly variable manifestation, from a mild phenotype to early-onset and rapidly progressive MFS. Cardiovascular abnormality could be the only defect in some affected individuals. According to the 2010 revised Ghent criteria, in the absence of family history, the combination of aortic root dilation (*Z* ≥ 2)/dissection and identification of a causal *FBN1* mutation was sufficient to establish a diagnosis of MFS [9]. Accordingly, we performed an MLPA assay to screen for *FBN1* and *TGFBR2* large genomic rearrangements not only in the diagnosed/suspected MFS patients but also in those early-onset aneurysm/dissection patients with minor skeletal and ocular involvement, who had a negative result in a 15-gene panel testing associated with heritable aortopathy.

Finally, five patients with large *FBN1* deletions were identified in our cohort. All five patients had multiple systemic deformities, and three of them met the 2010 Ghent criteria when genetic results were not available, while the other two met the diagnostic criteria until *FBN1* gross deletions were detected, probably due to their young ages. Meanwhile, no gross deletions/duplications were identified in patients with only aortic aneurysm/dissection but without other systemic involvement. This result supported the hypothesis that *FBN1* gross deletions usually lead to classic MFS [10–12].

Although gross genomic rearrangement within the *FBN1* gene only contributed to a small proportion of MFS genetic causes (1.8–2.9%) (UMD, http://www.umd.be/FBN1/; HGMD, http://www.hgmd.cf.ac.uk/ac/gene.php?gene=FBN1), it was important to identify the pathogenic mutation to afford the patient an opportunity for prenatal testing and preimplantation genetic diagnosis (PGD). *FBN1* mutations could be identified by sequencing in most Marfan patients (up to 93% in classic Marfan patients) [13]. However, Sanger sequencing and next-generation sequencing are commonly used in clinical genetic testing and are limited in their ability to detect large deletions and duplications. MLPA is a commonly used method to screen large del/dup, commercially, easily, and rapidly. In our cohort, 5 out of 62 patients (8.1%) with diagnosed or suspected MFS but with negative results in panel sequencing had large *FBN1* deletions, which proved it to be efficacious and cost-effective to screen for *FBN1* large genomic rearrangement in those MFS patients with multiple systemic involvements and a negative *FBN1* sequencing result.

Since MLPA and SNP (single-nucleotide polymorphism) arrays are more applicable in clinical genetic testing, increasing gross *FBN1* genomic deletions/duplications has been reported (summarized in Table 4), but until now, there has been no definite and conclusive genotype-phenotype correlation. Current studies reveal that the whole gene deletion of *FBN1* did not lead to a more severe phenotype [12], and in-frame deletion involving exon 24–53 seemed to result in a high risk of early-onset and rapidly progressive form of MFS [11, 14–16].

**Table 4:** Overview of MFS cases with gross deletions in *FBN1* gene

Variation		Patient		Reference PMID (year)
Deletion (*FBN1* exon affected)	Affected domains	Age (y)	Phenotype in papers	
Single-exon deletion				
*FBN1*:g.46,701,985_46,728,871 (Ex1)	–	25	Classic MFS	17492313 (2002)
*FBN1*:Ex1	–	NA	Classic MFS	24501682 (2013)
*FBN1*:Ex1	–	NA	Classic MFS	24793577 (2014)
*FBN1*:Ex2	–	52	Classic MFS	11700157 (2001)
*FBN1*:Ex3	1st EGF-like	NA	MFS	21907952 (2011)
*FBN1*:Ex6	3rd EGF-like	49	Potential MFS	28842177 (2017)
*FBN1*:c.3603_3668 del (Ex29)	18th cbEGF-like	After birth	Neonatal MFS	10441700 (1999)
*FBN1*:Ex30	19–20th cbEGF-like	< 1	Suspected Beals-Hecht syndrome	25944730 (2015)
*FBN1*:Ex32	21–22th cbEGF-like	1	Neonatal MFS	18412115 (2008)
*FBN1*:Ex36	25–26th cbEGF-like	NA	Classic MFS	19839986 (2009)
*FBN1:*g.48,749,026_48,753,819 del (Ex43)	7th TB, 29th cbEGF-like	24	Classic MFS	In this study
*FBN1*:g.48,734,801-48,730,690 del (Ex50)	35th cbEGF-like	14	MFS	In this study
*FBN1*:Ex52	8th TB, 36th cbEGF-like	40	Classic MFS	11700157 (2001)
*FBN1*:g.48,727,672-48,726,338 del (Ex54)	37–38th cbEGF-like	5	MFS	In this study
*FBN1:*g.48,724,560_48,722,281 del (Ex56)	39–40th cbEGF-like	38	Classic MFS	In this study
Multi-exon deletion				
*FBN1*:Ex1–5	1–3rd EGF-like	27	Classic MFS	21936929 (2011)
*FBN1*:g.46,580,456_46,883,035 (Ex1-16)	1–3rd EGF-like, 1st TB, 4–10th cbEGF-like	40	Classic MFS	17492313 (2002)
*FBN1*:Ex1–36	1–3rd EGF-like, 4–26th cbEGF-like, 1–5th TB	15	Classic MFS	28842177 (2017)
*FBN1*:g.48,890,962_48,922,918 (Ex2-4)	1–2nd EGF-like	32	Classic MFS	29850152 (2018)
*FBN1*:Ex6–65	3rd EGF-like, 4–47th cbEGF-like, 1–9th TB	NA	Classic MFS	24793577 (2014)
*FBN1*:Ex13–49	7–34th cbEGF-like, 3–7th TB	5	MFS	18412115 (2008)
*FBN1*:Ex24–26	14–16th cbEGF-like	After birth	Neonatal MFS	20455198 (2010)
*FBN1*:Ex33–38	21–26th cbEGF-like, 6th TB	1	Neonatal MFS	24199744 (2014)
*FBN1*:Ex34–43	23–29th cbEGF-like, 6–7th TB	22	Classic MFS	19863550 (2010)
*FBN1*:Ex37–65	26–47th cbEGF-like, 3–9th TB	NA	Classic MFS	24793577 (2014)
*FBN1*:Ex42–43	7th TB, 29th cbEGF-like	> 46	Classic MFS	11710961 (2001)
*FBN1*:Ex44–46	29–31th cbEGF-like	> 6	Childhood onset MFS	11710961 (2001)
*FBN1*:Ex44–66	29–47th cbEGF-like, 8–9th TB	37	Classic MFS	In this study
*FBN1*:Ex48–53	33–37th cbEGF-like, 8th TB	15	Neonatal MFS	28842177 (2017)
*FBN1*:Ex49–50	34–35th cbEGF-like	3	Neonatal MFS	28842177 (2017)
*FBN1*:Ex50–63	35–46th cbEGF-like, 8–9th TB	65	MFS	19659760 (2009)
*FBN1*:Ex58–63	41–46th cbEGF-like	17	Juvenile onset classic MFS	17189636 (2007)
*FBN1*:c.7456_7821 del^*^ (Ex61–64)	43–46th cbEGF-like	48	Classic MFS	1631074 (1994)
Whole gene deletion				
*FBN1*:Ex1–66	Full gene	16	Incomplete MFS	20478419 (2010)
*FBN1*:Ex1–66	Full gene	42	Classic MFS	21936929 (2011)
*FBN1*:Ex1–66	Full gene	15	Classic MFS	21936929 (2011)
*FBN1*:Ex1–66	Full gene	12	Classic MFS	21936929 (2011)
*FBN1*:Ex1–66	Full gene	41	MFS	21063442 (2011)
*FBN1*:Ex1–66	Full gene	39	MFS	21063442 (2011)
*FBN1*:Ex1–66	Full gene	16	MFS	21063442 (2011)
*FBN1*:Ex1–66	Full gene	13	MFS	21063442 (2011)
*FBN1*:Ex1–66	Full gene	27	MFS	21063442 (2011)
*FBN1*:Ex1–66	Full gene	21	MFS	21063442 (2011)
*FBN1*:Ex1–66	Full gene	34	MFS	21063442 (2011)
*FBN1*:Ex1–66	Full gene	5	Potential MFS	21063442 (2011)
*FBN1*:Ex1–66	Full gene	13	Potential MFS	21063442 (2011)
*FBN1*:Ex1–66	Full gene	8	Potential MFS	21063442 (2011)
*FBN1*:Ex1–66	Full gene	13	Classic MFS	22260333 (2012)
*FBN1*:g.48,931,968_51,102,375 (Ex1–66)	Full gene	14	MFS	27615407 (2016)

*NA* not available

*The deletion was represented as nt. 4762_5127 in partial cloned sequence of *FBN1* (PMID:1852207), and it was converted into its standardized nomenclature in accordance with HGVS (Human Genome Variation Society), in which the position + 1 corresponds to the A of the ATG start codon of the mRNA reference sequence (GenBank NM_000138) at the cDNA (c) level. Except for this, all of the other nucleotide positions and patient phenotypes were shown as it was reported in the reference article

## Conclusions

In summary, our data expand the number of large *FBN1* deletions and emphasize that screening for gross deletions in *FBN1* genes is necessary for clinically suspected MFS patients, especially in those who have a negative result in conventional sequencing methods.

## Methods

### Participants

Patients with MFS or early-onset aortopathy were referred for a genetic test from the Center of Vascular Surgery in Fuwai Hospital and Department of Cardiovascular Surgery in Xiangya Hospital Central South University. Of these, 115 patients in whom no causal mutation was identified in a 15-gene panel associated with heritable aortopathy, including *ACTA2*, *CLO3A1*, *FBN1*, *FBN2*, *MYH11*, *MYLK*, *NOTCH1*, *PRKG1*, *SKI*, *SLC2A10*, *SMAD3*, *SMAD4*, *TGFB2*, *TGFBR1*, and *TGFBR2*, were enrolled in this study to screen for *FBN1* and *TGFBR2* large del/dup.

### Multiplex ligation-dependent probe amplification (MLPA)

MLPA assays were performed to detect *FBN1* and *TGFBR2* large deletions or duplications using the commercially available SALSA MLPA kits P065 and P066 (MRC-Holland, Amsterdam, The Netherlands), which contained probes for all exons of *FBN1* and *TGFBR2*. According to the manufacturer’s instructions, a total of 100–200 ng of genomic DNA of each patient was used for hybridization, and amplification products from each MLPA assay were separated by capillary electrophoresis on an ABI 3500XL Dx Genetic Analyzer (Life Technologies, USA). The results were analyzed using Coffalyser software. Deletions and duplications with deviations more than 30% were suspected as significant alterations.

### Sanger sequencing

To verify the results of MLPA and identify the breakpoints of the deletions, we performed a long-range PCR and subsequent Sanger sequencing. Primers flanking the predicted deletions were designed and LA Taq Hot Start Version kit (Takara, Japan) was used in the PCR system with the following cycling process: 5-min initial denaturation at 96 °C, 30 cycles of 10 s at 98 °C, and 15 min at 68 °C, finished by a 10-min final extension step at 72 °C. Then, the products were detected through agarose gel electrophoresis and sequenced by the inner primers on the ABI 3730XL Genetic Analyzer.

### Quantitative PCR

Quantitative PCR (qPCR) with the SYBR green reporter dye was performed to quantify relative target gene regions copy number in genomic DNA, and housekeeping gene *GAPDH* (glyceraldehyde 3-phosphate dehydrogenase) was used as the reference gene. The primer pairs were designed by Primer3 Input (http://bioinfo.ut.ee/primer3-0.4.0/) (see Additional file 1). All qPCRs were performed using 2 × SYBR FAST qPCR Kit Master Mix (KAPA Biosystems, America) with the QuantStudio 6 Flex Real-Time PCR System.

## Additional file

Additional file 1: **Table S1.** The quantitative PCR primer pairs for AD437. (PDF 132 kb)

## Abbreviations

del: Deletion
dup: Duplication
*FBN1*: Fibrillin-1
*GAPDH*: Glyceraldehyde 3-phosphate dehydrogenase
HGMD: Human Gene Mutation Database
HGVS: Human Genome Variation Society
LDS: Loeys-Dietz syndrome
MFS: Marfan syndrome
MLPA: Multiplex ligation-dependent probe amplification
NA: Not available
qPCR: Quantitative PCR
SNP: Single-nucleotide polymorphism
UMD: Universal Mutation Database
