# Supplementary material for: Identification of gross deletions in FBN1 gene by MLPA
Source: Hum Genomics. 2018 Oct 4;12:46. doi: 10.1186/s40246-018-0178-y (PMC6172713; doi:10.1186/s40246-018-0178-y)
Supplement: Supplementary file 1 — Table S1. The quantitative PCR primer pairs for AD437. (PDF 132 kb) [file 40246_2018_178_MOESM1_ESM.pdf]

Table S1. The quantitative PCR primer pairs for AD437

Primer target	Primer name	Primer sequence	Product size
FBN1-EX55	EX55-F	TAAATGAATGTGCCCAGAATCC	139bp
	EX55-R	CTTGAACACGATGACTCACCTT	
FBN1-EX66	EX66-F	CGAATCACAACAGATACTTGATCG	228bp
	EX66-R	TCTTCAGATTATCACCCAGTTCAC	
GAPDH	GAPDH-Q-F	CTATAAATTGAGCCCGCAGCC	152bp
	GAPDH-Q-R	GCCCAATACGACCAAATCCGT	
